# Fatal outcome of posterior reversible encephalopathy syndrome (PRES) in a lupus nephropathy patient: A case report

**DOI:** 10.1016/j.radcr.2022.03.084

**Published:** 2022-04-19

**Authors:** Ihssane Afilal, Siham Nasri, Mustapha Bendaoud, Hajar Mahjouba, Imane Guerrouj, Fathia Aidid, Widad Abbou, Narjisse Aichouni, Imane Kamaoui, Imane Skiker

**Affiliations:** Department of Radiology, Mohammed VI University Hospital, Faculty of Medicine, University Mohammed First, BP 4806 Oujda University, Oujda 60049, Morocco

**Keywords:** Posterior reversible encephalopathy syndrome (PRES), Systemic lupus erythematosus disease (SLED), Lupus nephropathy, Seizure, Hypertension, MRI

## Abstract

Posterior reversible encephalopathy syndrome is a rare underestimated condition, that generally complicates a rise in blood pressure in an acute setting. This entity has been increasingly identified in patients with systemic lupus erythematosus disease. PRES is challenging to diagnose seeing as it presents with nonspecific neurological symptoms, such as head-aches, confusion, seizures, visual changes or a coma, and can mimic neuropsychiatric lupus. Imaging plays a necessary role in confirming this diagnosis, as it is characterized by vasogenic edema of the posterior white matter, in which the distribution is bilateral and symmetrical. Although this syndrome is rare, early diagnosis allows a prompt treatment and therefore a favorable outcome. We present a case report of PRES in a 14-year-old female previously diagnosed with lupus nephropathy, who presented to the emergency department with seizures and uncontrolled hypertension, that was unfortunately not reversible is this patient.

## Introduction

Posterior reversible encephalopathy syndrome (PRES), previously known as acute hypertensive encephalopathy or reversible posterior leukoencephalopathy [1]. These terms may be a misnomer; as lesions are not always located posteriorly and reversibility is not always present, even though most cases describe a resolution of symptoms after adequate management, some cases of fatal outcome or irreversible neurological damage following PRES have been reported. This neurological deficit is also widely misdiagnosed, and presents with nonspecific symptoms, including headaches, seizure, altered mental state, visual disturbance, ataxia, vertigo, or even a coma [2]. Although the exact etiology of PRES is not fully understood yet, it has been reported that it could be due to vasogenic edema caused by loss of auto-regulation mechanisms, or endothelial dysfunction, in response to sudden changes in blood pressure [3]. Risk factors include severe hypertension, acute glomerulonephritis, auto-immune disorders, such as systemic lupus erythematosus (SLED), eclampsia, preeclampsia, sepsis, haemolytic uremic syndrome, and cytotoxic drugs [4]. The prevalence of PRES in patients with SLED has been recorded in 0.43% in a case-control study [3], Although rare among SLED patients, PRES tends to be associated with high mortality rates and mainly concerns female gender [5]. A study has shown that the systemic Lupus Erythematosus Disease Activity Index criteria for lupus was higher than 6 points in patients that developed PRES, indicating a higher severity of disease at the time of onset [6].

The diagnosis is mainly radiological, computed tomography (CT) may visualize hypodense lesions, but magnetic resonance imaging (MRI) remains the gold standard for diagnosis and shows typical images of hyperintensities on T2 and fluid-attenuated inversion recovery (FLAIR) and hypointensities on T1. The topography is predominantly bilateral and symmetrical in the posterior parieto-occipital lobes, but can also be unilateral and affect other areas of the brain [3], including frontal, inferior temporal, cerebellar, and brainstem regions [7].

This case highlights the importance of considering this potentially reversible syndrome, in young women with or without a pre-established risk factor of PRES, presenting to the emergency room with neurological symptoms in an acute setting, as well as the necessity of early stage diagnosis and timely management in order to avoid irreversible damage.

## Patient and observation

**Fig. 1:**
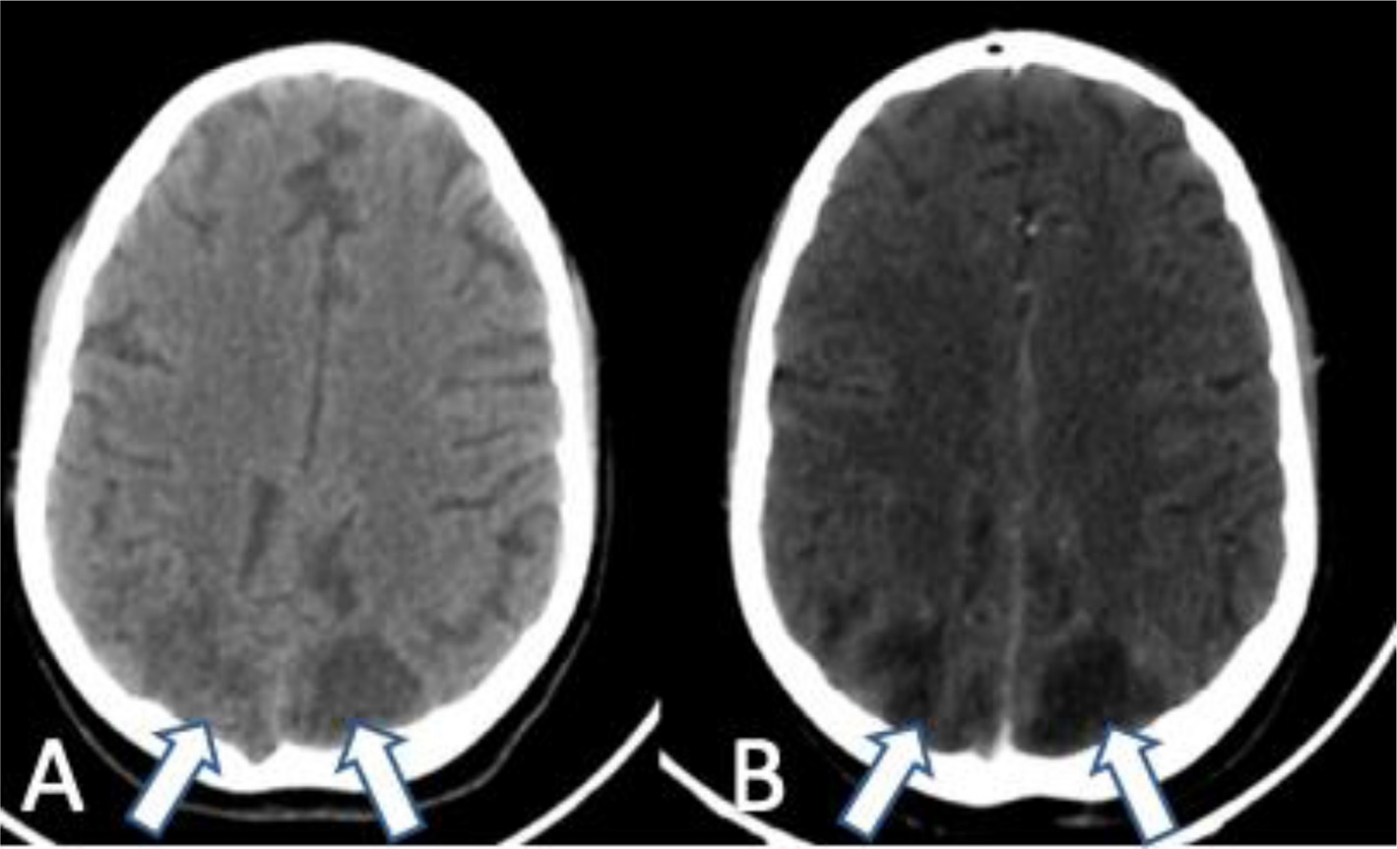
Axial CT obtained day 1 of admission, showing subtle bilateral, grossly symmetrical, cortical and sub-cortical posterior parietal hypodensities (arrows), unchanged before (A) and after contrast (B), consistent with PRES.

A 14-year-old female patient, with a pre-established diagnosis of lupus nephropathy, suspected clinically with an array of symptoms, such as prolonged fever, recurring infections, arthritis and photo-sensibility, diagnosed biologically (Table 1) with positive antinuclear antibodies, and confirmed histologically with a renal biopsy. The patient was prescribed mycophenolic acid 500 mg/day and prednisolone 40 mg/day. She has also received 2 doses of coronavirus disease 2019 messenger RNA vaccines. The patient has been experiencing headaches 3 days before she was admitted to the emergency department with a first episode of seizure. Clinical examination showed Glasgow coma scale was at 12/15, sever hypertension at 190/117 mmHg, tachycardia of 150 beats per minute, oxygen saturation on ambient air was 98%, she also presented generalized edema and a urinary tract infection. Moreover, she had no meningeal signs, and metabolic causes were eliminated. A CT scan (Fig. 1) was initially ordered and showed hypodense posterior parietal lesions that were bilateral, grossly symmetrical, unsystematized and unenhanced after contrast

**Table 1:** Laboratory findings.

Labs	Value	Reference range
Creatinine	18.47	6-13 mg/L
Potassium	5.6	3.0-5.0 mmol/L
ESR	116	0-20 mm/h
Hemoglobin	7.2	12-16 g/dL
WBC count	28.12	4.0-10.0 thousand/mm^3^
CRP	58	<5 mg/L
Procalcitonin	5.9	<0.1 ng/mL
ANA	Positive	—

**Fig. 2:**
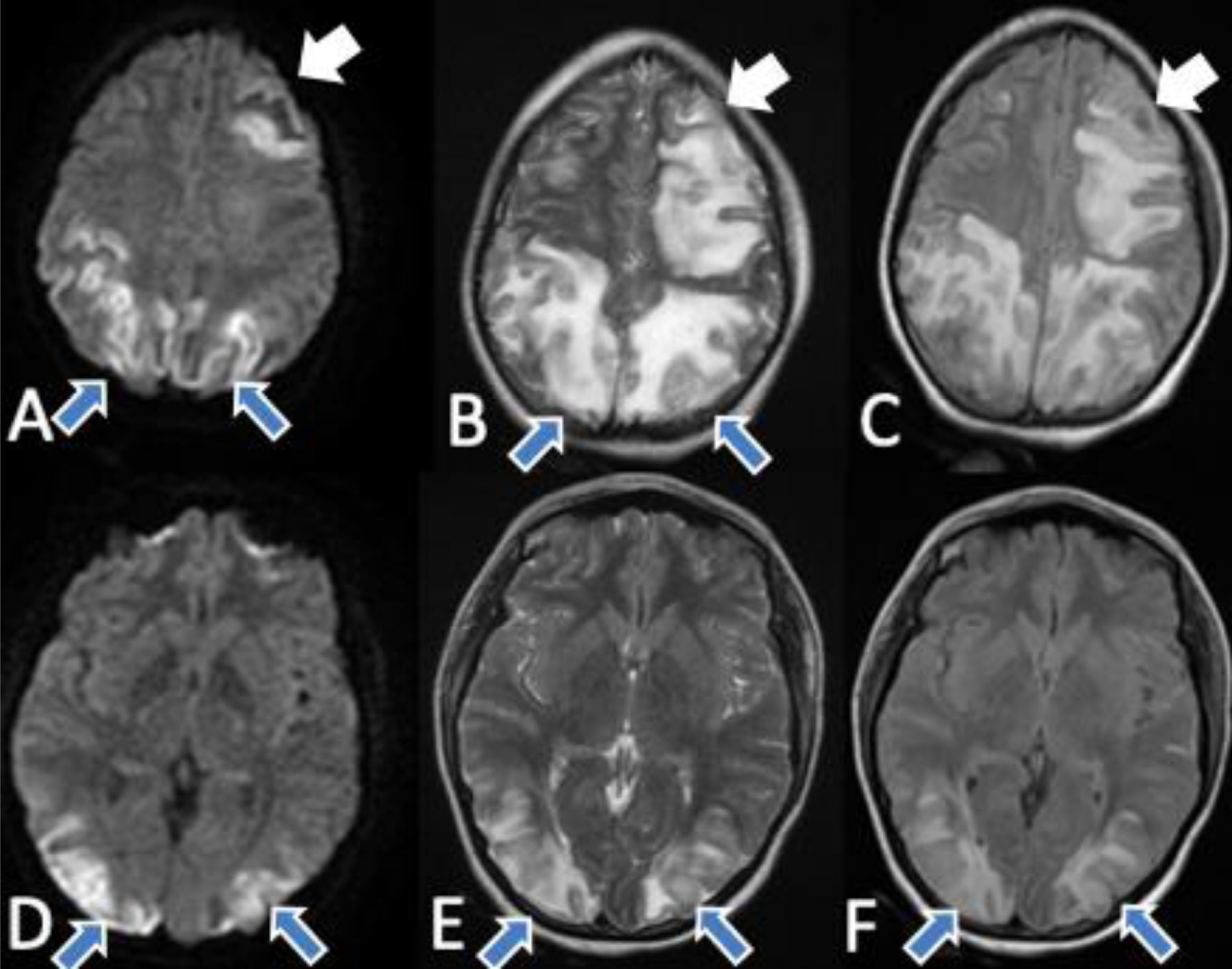
Brain MRI obtained on day 2 of admission, demonstrating extensive vasogenic edema with few areas of ischemic changes, in frontal (white arrows), parietal and occipital lobes (blue arrows), in diffusion sequences: (A-D), T2 sequences: (B-E), FLAIR sequences: (C-F) (Color version of figure is available online).

An MRI (Fig. 2) was performed to further confirm the diagnosis and eliminate the differentials. It showed signal anomalies of the frontal and parieto-occipital cortical and sub-cortical white matter with increased signal intensity in T2 sequences, and high signal intensity on the apparent diffusion coefficient. Based on these clinical and radiological findings we were able to confirm the diagnosis of PRES.

Anti-hypertensive treatment was reinforced, immunosuppressive therapy was discontinued and replaced with a dose of 36 mg/day of prednisone, as well as antibiotics to treat the urinary tract infection. The patient's neurological symptoms worsened and the intensive care unit doctors had to intubate and sedate her. The patient's condition progressively deteriorated while she remained in the intensive care unit for the next week, at which time hypotension and bradycardia resulted in cardiac arrest from which she could not be resuscitated.

## Discussion

PRES is a rare and underrecognized syndrome, previously called reversible posterior leukoencephalopathy when it was first described as a distinct entity in 1996 by an American neurologist Judy Hinchey et al [10]. The term PRES is somewhat of misnomer considering there are cases of irreversible damage or even fatal outcome, and this syndrome can extend beyond the posterior regions of the brain. It is a clinical and radiological entity, associating a pathognomonic triade, characterized with neurological symptoms, typical imaging findings and a rapidly reversible evolution as soon as the causing factor is managed.

The physiopathology of PRES in SLED is not yet completely explained, however 2 theories have been reported, that are not necessarily mutually exclusive [7]. The first one is the breakthrough theory [1] of encephalic hyperperfusion due to a transitory and severe elevation in blood pressure, leading to disruption of the brain autoregulation system and eventually in vasogenic edema, which explains the rapid reversibility once blood pressure is controlled at an early stage. The second theory is a reflexe hypopefusion of the brain secondary to endothelial dysfunction and exacerbated by vasoconstriction, spasm and/or auto-immune activation, therefore resulting in vascular hyperpermeability and vasogenic edema. All these conditions may be coinciding, creating a summative or multiplicative effect on the course of the disease [11].

Clinically it presents with neurosensory impairment such as headaches, confusion, agitation, visual disturbance, seizures or even coma. PRES tends to concern more women than men. Risk factors are numerous, including hypertension, eclampsia, auto-immune diseases, such as SLED, renal dysfunction, thrombocytopenic thrombotic purpura, hemolytic-uremic syndrome, immunosuppressive drugs, bone marrow/stem cell/organ transplantation, sepsis, hyperamonemia, sickle cell disease [8] and even ventriculo-peritoneal shunt insertion/overshunting [9]. A lumbar puncture could be useful to eliminate meningitis, or a malignant cause. However, cerebrospinal fluid in PRES often only shows moderately elevated protein levels with less than 5 leucocytes/µl [12]. Differential diagnosis for PRES in a patient with a pre-established diagnosis of SLED, includes lupus cerebritis, neuropsychiatric-lupus, meningitis, ischemia, hemorrhage, a primary seizure disorder and many others, and PRES should be considered an elimination diagnosis with supportive findings.

CT imaging is usually ordered first in an emergency setting, to rule out the differentials, and it can visualise subtle hypodensities consistent with PRES. However, MRI is considered the gold standard for the diagnosis. It shows a vasogenic edema of the cortical and sub-cortical white matter, in which the distribution is predominantly nonvascular, bilateral and symmetrical in the posterior parieto-occipital lobes, but can also be unilateral and affect other regions of the brain [3] including frontal, inferior temporal, cerebellar, and brainstem regions [7]. These lesions are typically hyperintense on T2 and FLAIR sequences, and hypointense in T1 and diffusion-weighted imaging, with a high signal on apparent diffusion coefficient. Diffusion-weighted imaging is mainly useful to differentiate PRES from ischemia, as we find low intensity signal lesions in PRES consistent with vasogenic edema, and high intensity signal lesions in cerebral ischemia consistent with cytotoxic edema.

The treatment of PRES remains symptomatic and etiological, and consists of rapid maintain of blood pressure and neuro-reanimation to avoid permanent damage. Patients presenting with PRES who are also on immunosuppressive therapy should have their medication temporarily discontinued or a decrease in dosage until the patient improves [12]. The prognosis in usually favorable in the classic form of PRES, as there is complete regression of the clinical and radiological signs once the underlying cause is managed promptly, nevertheless this reversible outcome is not always found. Death is usually linked to neurological complications and a delay in time of diagnosis and management.

## Conclusion

PRES is a largely misdiagnosed and underestimated reversible neurological deficit and should be considered in all young women presenting with neurological symptoms, especially in a context of mismanaged hypertension, active SLED, uncontrolled nephropathy or other risk factors. MRI FLAIR sequence is the gold standard diagnostic method, PRES lesions are typically diffuse symmetrical posterior T2 and FLAIR hyperintensities of the white matter. Given the good prognosis of PRES, early diagnosis allows a prompt and adapted treatment and therefore a favorable outcome by preventing permanent neurological deficits or a fatal outcome.

## Funding

No funding was received for this work.

## Patient consent

The authors certify that they have obtained all appropriate patient consent forms. In the form the patient(s) has/have given his/her/their consent for his/her/their images and other clinical information to be reported in the journal. The patients understand that their names and initials will not be published and due efforts will be made to conceal their identity, but anonymity cannot be guaranteed.

## References

[1] Gaillard, F, Luong, D Posterior reversible encephalopathy syndrome. Reference article (2008). doi:10.53347/rID-1915.

[2] Ellis CA, McClelland AC, Mohan S, Kuo E, Kasner SE, Zhang C, Khankhanian P, Balu R. (2019). Cerebrospinal fluid in posterior reversible encephalopathy Syndrome: Implications of elevated protein and pleocytosis. Neurohospitalist.

[3] Vaysman T, Xu P, Vartanian T, Michalak P, Pike K, Liu A (2019). “Highlighting” red nuclei by atypical posterior reversible encephalopathy syndrome in a patient with systemic lupus erythematosus. Clin Case Rep.

[4] Merayo-Chalico Javier (2016). Clinical outcomes and risk factors for posterior reversible encephalopathy syndrome in systemic lupus erythematosus: a multicentric case–control study. J Neurol Neurosurg Psychiatry.

[5] Mikdashi J, Nived O (2015). Measuring disease activity in adults with systemic lupus erythematosus: the challenges of administrative burden and responsiveness to patient concerns in clinical research. Arthritis Res Ther.

[6] Damrongpipatkul U, Oranratanachai K, Kasitanon N, Wuttiplakorn S, Louthrenoo W (2018). Clinical features, outcome, and associated factors for posterior reversible encephalopathy in Thai patients with systemic lupus erythematosus: a case-control study. Clin Rheumatol.

[7] Tetsuka S, Ogawa T (2019). Posterior reversible encephalopathy syndrome: a review with emphasis on neuroimaging characteristics. J Neurol Sci.

[8] Thust SC, Burke C, Siddiqui A (2014). Neuroimaging findings in sickle cell disease. Br J Radiol.

[9] Merola J, Magdum S (2017). An unusual complication following ventriculoperitoneal shunting. J Pediatr Neurosci.

[10] Hinchey J, Chaves C, Appignani B, Breen J, Pao L, Wang A (1996). A reversible posterior leukoencephalopathy syndrome. N Engl J Med.

[11] Hartman EN, Tuna K, Monsour E, Komanduri K, Tarasiuk-Rusek A (2020). Seizure as an initial presentation for posterior reversible encephalopathy syndrome in undiagnosed systemic lupus erythematosus and lupus nephritis: a case report. Cureus.

[12] Jabrane M, Ait Lahcen Z, Fadili W, Laouad I (2015). A case of PRES in an active lupus nephritis patient after treatment of corticosteroid and cyclophosphamide. Rheumatol Int.

